# Systemic atrioventricular valve replacement due to a supravalvular stenosing ring and ebsteinoid tricuspid valve in a patient with congenitally corrected transposition of the great arteries: a case report

**DOI:** 10.47487/apcyccv.v6i4.522

**Published:** 2025-12-29

**Authors:** Nathalie Victoria Zacarías Mendoza, Milagros Elizabeth Napán Herrera, Diana Vanessa Carassa Rodríguez, Víctor Justo Robles Velarde

**Affiliations:** 1 Facultad de Medicina «Alberto Hurtado», Universidad Peruana Cayetano Heredia, Lima, Perú. Universidad Peruana Cayetano Heredia Facultad de Medicina «Alberto Hurtado» Universidad Peruana Cayetano Heredia Lima Peru; 2 Servicio de Cirugía Cardiovascular, Instituto Nacional Cardiovascular - INCOR, Lima, Perú. Servicio de Cirugía Cardiovascular Instituto Nacional Cardiovascular - INCOR Lima Perú

**Keywords:** Congenitally Corrected Transposition of the Great Vessels, Congenital heart disease, Tricuspid Valve Stenoses, Ebstein´s Anomaly, Transposición Congénitamente Corregida de las Grandes Arterias, Cardiopatías Congénitas, Estenosis de la Válvula Tricúspide, Anomalía de Ebstein

## Abstract

Congenitally corrected transposition of the great arteries (ccTGA) is a rare and complex cardiac malformation often associated with additional anomalies. We present a 23-year-old patient with ccTGA, a supravalvular stenosing ring, and an Ebsteinoid tricuspid valve who developed severe systemic atrioventricular valve (SAVV) stenosis and regurgitation. Preoperative evaluation revealed tricuspid valve abnormalities, a dilated systemic right ventricle (sRV) with a reduced sRV ejection fraction (42%). The patient underwent bioprosthetic SAVV replacement and excision of the supravalvular membrane. Postoperatively, valve function was preserved, and recovery was favorable despite transient complications. This case emphasizes the surgical challenges and individualized decision-making required in ccTGA with rare anatomical variants, highlighting the value of timely intervention, multidisciplinary care, and long-term follow-up to optimize outcomes.

## RESUMEN

La transposición congénitamente corregida de las grandes arterias (TCCGA) es una malformación cardíaca rara y compleja, frecuentemente asociada con otras anomalías estructurales. Se presenta el caso de un paciente de 23 años con TCCGA, un anillo estenosante supravalvular y una válvula tricúspide ebsteinoide, que desarrolló estenosis e insuficiencia severas de la válvula auriculoventricular sistémica (VAVS). La evaluación preoperatoria mostró anomalías tricuspídeas significativas, dilatación del ventrículo derecho sistémico (VDS) y fracción de eyección reducida (42%). Se realizó reemplazo bioprotésico de la VAVS y resección de la membrana estenosante supravalvular. En el posoperatorio, la válvula mostró adecuada funcionalidad y el paciente tuvo evolución favorable pese a complicaciones transitorias. Este caso resalta los desafíos quirúrgicos y la necesidad de un abordaje multidisciplinario en la TCCGA con variaciones anatómicas inusuales, subrayando la importancia de la intervención oportuna, el seguimiento prolongado y la atención individualizada para optimizar los resultados.

## Introduction

Congenitally corrected transposition of the great arteries (ccTGA) is a rare and complex congenital malformation whose management remains challenging. ^1^^-^^3^ This extremely rare entity (approximately 0.05% of congenital heart malformations, with an incidence of approximately 1 in 33,000 live births) ^2^ and the complexity of ccTGA have hindered the development of a universally acceptable treatment strategy. In ccTGA, additional anomalies are present in 99% of cases, including atrial septal defects (ASD), while ventricular septal defects (VSD) are found in 90% of cases. Additionally, pulmonary valve (PV) stenosis or atresia is present in 65% of cases, and tricuspid valve (TV) abnormalities, functioning as the systemic atrioventricular valve (SAVV), are noted in 46%, anatomically similar to Ebstein anomaly.(1) These abnormalities can impact the patient’s clinical presentation. ^1^^,^^4^

Most adult patients develop symptoms as progressive dysfunction of the systemic ventricle leads to systemic atrioventricular valve (SAVV) regurgitation. In ccTGA, the AV conduction axis is located anterior-superiorly, which increases susceptibility to both spontaneous and surgically induced heart block. Surgical management becomes necessary for patients presenting with systemic ventricular dysfunction, while early intervention may also be warranted in the presence of associated anomalies. These may include replacement of the SAVV, closure of atrial or ventricular septal defects, and correction of abnormal pulmonary venous return. ^2^^,^^3^ We report a rare case of ccTGA with a supravalvular stenosing ring and an Ebsteinoid tricuspid valve, which posed challenges in determining the most appropriate surgical intervention.

## Case report

**Figure 1:**
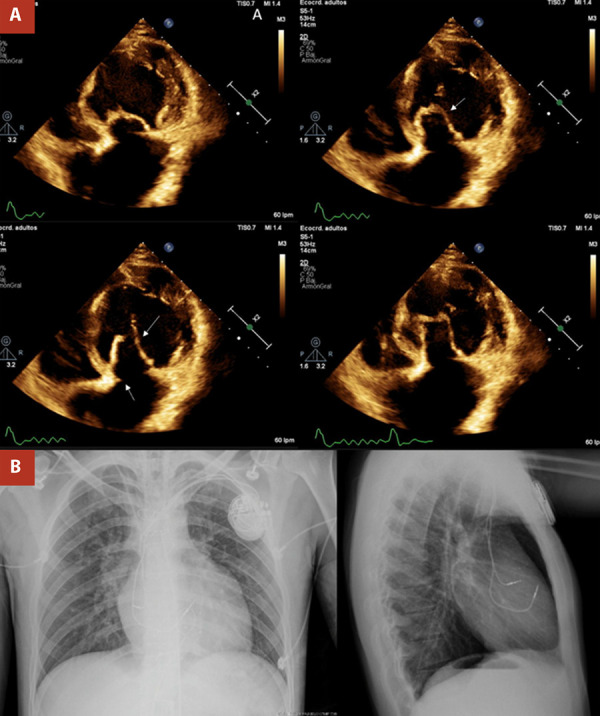
(A) Two-dimensional and Doppler echocardiogram. Arrows mark an Ebsteinoid systemic atrioventricular valve with severe insufficiency and a supravalvular stenosing ring. (B) Chest X-ray demonstrates pacemaker leads in the right heart chambers.

A 23-year-old man with ccTGA, diagnosed at the age of 1 month, was referred to our institution for surgical treatment of severe tricuspid stenosis due to a supravalvular ring, coupled with severe tricuspid regurgitation associated with an Ebsteinoid tricuspid valve. Moreover, he underwent dual-chamber pacemaker placement at the age of 12 due to third-degree atrioventricular block, which was replaced 8 years later. He also received chronic anticoagulation therapy for permanent atrial fibrillation. The patient was asymptomatic until 2019, the year in which the surgery was scheduled but postponed for unspecified reasons. At the beginning of 2022, the patient reported exertional dyspnea associated with palpitations. Two-dimensional and Doppler echocardiogram revealed ccTGA with a dilated systemic right ventricle (sRV) and decreased systolic function (fractional area change (FAC): 24%). The SAVV was dysplastic with severe insufficiency, and a supravalvular ring caused significant stenosis (mean gradient: 9 mmHg, maximum velocity: 2.2 m/s). The subpulmonary AV valve showed mild insufficiency, while the subpulmonary ventricle had normal dimensions and preserved systolic function (ejection fraction: 53%). Pacemaker leads were present in the right heart chambers (Figure 1).

**Figure 2:**
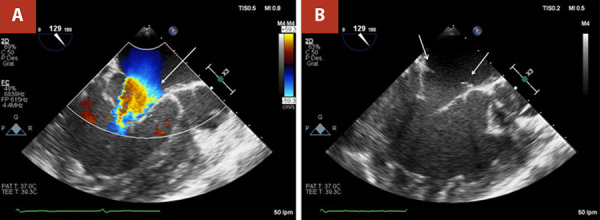
Two-dimensional preoperative transesophageal echocardiography (2D-TEE) with and without color Doppler. **(A)** 2D-TEE three-chamber view showing turbulent flow (white arrow) and **(B)** Supravalvular stenosing ring (white arrows) in the systemic atrioventricular valve at 87 degrees.

The initial vital signs were normal (heart rate was 62 beats/min, blood pressure was 100/70 mmHg, respiratory rate was 18/min with an oxygen saturation of 97%, and temperature was 36.5 °C). Physical examination revealed regular heartbeats with a mild systolic murmur of mitral regurgitation. New York Heart Association (NYHA) was Class II. Laboratory evaluation was normal. Preoperative two-dimensional transesophageal echocardiography (2D-TEE) revealed ccTGA with a dilated sRV and a reduced sRV ejection fraction (sRVEF, 42%) and diffuse hypokinesis, a subpulmonary ventricle of normal dimensions and preserved ejection fraction (53%), and severe dilation of the left atrium. In addition, we observed severe SAVV regurgitation associated with an Ebsteinoid valve, showing mild apical displacement of the septal leaflet relative to the mitral annulus, severe tricuspid stenosis (area: 1.5 cm^2^; maximum gradient: 15 mmHg; mean gradient: 7 mmHg) due to a supravalvular ring, mild pulmonary AV valve regurgitation, and pacemaker leads in the right cavities (Figure 2).

The patient underwent bioprosthetic replacement of the SAVV, left atrial appendage closure, and excision of a supravalvular stenosing membrane through median sternotomy. The surgery, using central arterial and bicaval venous cannulation, clamping, and cardioplegic arrest using Custodiol solution, revealed a dysmorphic SAVV with severe leaflet prolapse, a dilated 30 mm ring, a supravalvular membrane, and mesocardia of ccTGA. The membrane was excised, and a 27 mm bioprosthetic valve (Hancock™ II T510, Medtronic, Minneapolis, MN, USA) was implanted with native leaflet retraction. A bioprosthetic valve was chosen instead of a mechanical one because it was the only option covered by the patient’s insurance. Closure and extracorporeal circulation were complication-free, leading to ICU transfer for recovery.

Postoperative TEE demonstrated the functional integrity of the bioprosthetic valve in the systemic position, showing no dysfunction (velocity: 1.5 m/s, mean gradient: 5 mmHg), no residual regurgitation, and no pericardial effusion. Mild PV regurgitation and severe systemic ventricular dysfunction (ejection fraction: 39%) were noted. In the immediate postoperative period, the patient received ventilatory support and a dobutamine infusion, and although he experienced transient respiratory and cardiac complications, he exhibited a favorable outcome. During the postoperative days, he received treatment that included management of thrombotic risk and heart failure. The treatment consisted of metamizole sodium 1 g as needed for pain, paracetamol 1 g, cefazolin 1 g, enalapril 5 mg, spironolactone 25 mg, furosemide 40 mg, ranitidine 300 mg, warfarin 2.5 mg, enoxaparin sodium 60 mg, carbamazepine 20 mg, and diazepam 10 mg as needed for seizures. Discharge occurred on the 12th day post-surgery without complications. At the subsequent follow-ups, he reported that his exertional dyspnea had resolved.

## Discussion

Patients with ccTGA who do not undergo anatomic correction in infancy may experience complications such as sRV dysfunction and SAVV regurgitation. ^5^ SAVV regurgitation emerges as a significant challenge in both operated and nonoperated ccTGA patients, becoming the primary factor in sRV failure and death. ^1^^,^^6^ Timely surgical referral is crucial to maintaining the durability of the physiologic repair strategy. ^7^ The mechanism behind SAVV regurgitation is multifactorial and not completely understood, involving factors such as congenital leaflet abnormalities (similar to Ebstein anomaly), RV or papillary muscle dysfunction, dilated TV annulus, dyssynchronous contraction (bundle branch block, complete heart block), and various arrhythmias. ^1^

Ebstein anomaly involves the displacement of the septal and mural leaflets of the tricuspid valve in the right ventricle (RV), leading to atrialization and a functionally hypoplastic RV. ^8^ According to Abdelrehim *et al*., intrinsic valve abnormalities are identified as the most prevalent cause of regurgitation (76.9%). Notably, “Ebsteinoid” is the most prevalent subclassification of valve abnormalities. ^7^ Unlike in concordant hearts, where Ebstein anomaly features include thinning of the atrialized portion of the RV and severe leaflet tethering, these features are generally absent in ccTGA. Therefore, the term “Ebsteinoid” is frequently used to describe the TV in ccTGA, highlighting its distinct morphological characteristics. ^1^

Tricuspid valve stenosis and, less commonly, the presence of a supravalvular stenosing ring are infrequently observed conditions in ccTGA. ^9^^-^^12^ Allwork *et al.* found SAVV abnormalities in 91% of the cases observed in their autopsy study of 32 cases of ccTGA, with only five cases showing a supravalvular stenosing ring. ^13^ Supratricuspid membranes share pathophysiological consequences similar to the supramitral ring in hearts with concordant atrioventricular and ventriculoarterial connections. ^14^

Surgical management of ccTGA involves two core principles: physiologic repair and anatomic repair. Physiologic repair focuses on the repair of concomitant defects to complete septation of the heart for an in-series biventricular repair without correction of atrioventricular and ventriculoarterial discordance. This strategy is typically reserved for patients with preserved sRV function and mild or absent SAVV regurgitation since it involves lower operative risk and technical complexity. However, long-term follow-up often reveals progressive sRV failure and SAVV regurgitation due to the chronic pressure load on the morphological right ventricle. Anatomic repair (double switch) aims to restore blood flow paths through the “normal” sequence of anatomic structures. This strategy is recommended for patients with evidence of sRV dysfunction or significant tricuspid regurgitation who are poor candidates for a purely physiologic approach. Although this repair offers superior long-term ventricular function, it is technically demanding, associated with longer cardiopulmonary bypass times, and carries a higher early operative risk. ^2^^-^^4^

Despite clear indications for anatomic repair, ^1^ including severe SAVV regurgitation and stenosis and a dilated sRV with a reduced sRVEF (42%), the decision-making process was significantly influenced by the available resources and capacities at the medical center. Confronted with restrictions in surgical capacity, the decision leaned toward prioritizing physiologic over anatomic correction. The choice was guided by the technical complexity and postoperative risks inherent in double-switch procedures, particularly in adult patients with long-standing ventricular remodeling, where surgical morbidity remains considerable.

Long-term care after SAVV replacement in ccTGA focuses on bioprosthesis durability, risk of reintervention, and sRV function. Bioprosthetic valves generally provide good hemodynamics but are susceptible to structural valve degeneration under systemic pressures; reintervention typically relates to leaflet degeneration, pannus, thrombosis, or endocarditis. Accordingly, surveillance should include transthoracic echocardiography at 1, 3, 6, and 12 months, and annually thereafter, assessing mean gradient/EOA, any prosthetic regurgitation, and sRV performance (fractional area change or volumetric indices, tissue Doppler/strain). Given the high prevalence of conduction disease and atrial arrhythmias in ccTGA, follow-up also includes rhythm monitoring and pacemaker checks, considering CRT when dyssynchrony contributes to sRV dysfunction. Functional status (NYHA class, 6-minute walk, natriuretic peptides) helps guide therapy optimization. ^1^ In our patient, we anticipate early symptomatic improvement and stable prosthetic gradients, but lifelong surveillance is required to detect bioprosthetic degeneration or progressive sRV dysfunction, which may ultimately prompt reintervention.

SAVV replacement, indicated in patients with severe symptomatic tricuspid regurgitation with an ejection fraction greater than 40% (Class I recommendation) ^15^, has been associated with improved survival and functional status, with NYHA Class I or II status ranging from 88% to 96% at median times of 4.7 to 16.9 years following replacement. ^4^^)^ Abdelrehim *et al.*^7^ highlight the value of SAVV surgery in ccTGA, with a low risk of early mortality (0.9%) and satisfactory late outcomes, despite 50% of the cohort being referred in an advanced stage with SVEF <40%. However, data on the long-term outcomes of SAVV surgery in ccTGA are currently limited. Overall, freedom from reintervention following physiologic repair or ccTGA is low. ^4^

Without surgery, the RV in ccTGA is burdened to generate systemic blood pressure, risking long-term failure and tricuspid valve regurgitation, which leads to progressive heart failure. Postponing surgery until age 22, our patient exhibited severe dilation of the left atrium, a dilated sRV with a reduced sRVEF (42%), and a New York Heart Association (NYHA) classification of Class II.

While previous reports have described isolated supravalvular rings or systemic atrioventricular valve regurgitation, few have documented the coexistence of both lesions in the same patient and the resulting technical and strategic implications for repair.

In conclusion, this case underscores the complexities in managing ccTGA, requiring a multidisciplinary approach and personalized surgical decisions based on individual anatomical and clinical characteristics. Balancing the principles of physiologic and anatomic repair presents challenges, emphasizing the crucial need to carefully evaluate each patient’s unique circumstances. Despite delayed surgical intervention, the patient’s post-surgery quality of life remains positive, emphasizing individualized patient management and ongoing monitoring. Further research and long-term follow-up studies are essential to advance understanding, refine treatment strategies, and contribute to improving outcomes for individuals facing the complexities of ccTGA.

## References

[1] Karl TR, Jacobs JP, Mavroudis C, Backer CL (2023). Congenitally corrected transposition of the great arteries [Internet].

[2] Kutty S, Danford DA, Diller GP, Tutarel O (2018). Contemporary management and outcomes in congenitally corrected transposition of the great arteries. Heart.

[3] Kumar TKS (2020). Congenitally corrected transposition of the great arteries. J Thorac Dis.

[4] Spigel Z, Binsalamah ZM, Caldarone C (2019). Congenitally corrected transposition of the great arteries anatomic, physiologic repair, and palliation. Semin Thorac Cardiovasc Surg Pediatr Card Surg Annu.

[5] Emani SM, Del Nido PJ (2023). Systemic atrioventricular valve regurgitation in corrected transposition of the great arteries: where are we?. J Am Coll Cardiol.

[6] Mulla S, Asuka E, Bora V, Siddiqui WJ (2023). Tricuspid regurgitation. In: StatPearls [Internet].

[7] Abdelrehim AA, Stephens EH, Miranda WR, Todd AL, Connolly HM, Egbe AC (2023). Systemic atrioventricular valve surgery in patients with congenitally corrected transposition of the great vessels. J Am Coll Cardiol.

[8] Singh DP, Hussain K, Mahajan K (2023). Ebstein anomaly and malformation. In: StatPearls [Internet].

[9] Bini RM, Favilli S, Murzi B, Moschetti R, Santoro G, Traini AM (2007). An unusual case of tricuspid lesion in congenital corrected transposition of the great arteries. J Cardiovasc Med.

[10] Marino B, Sanders SP, Parness IA, Colan SD (1986). Obstruction of right ventricular inflow and outflow in corrected transposition of the great arteries (S,L,L) two-dimensional echocardiographic diagnosis. J Am Coll Cardiol.

[11] Ritter M, Schneider J, Schmidlin D, Jenni R (1992). Supravalvular tricuspid ring and Ebsteinï¿1/2s anomaly in congenitally corrected transposition. Am J Cardiol.

[12] Chesler E, Beck W, Barnard CN, Schrire V (1973). Supravalvular stenosing ring of the left atrium associated with corrected transposition of the great vessels. Am J Cardiol.

[13] Allwork SP, Bentall HH, Becker AE, Cameron H, Gerlis LM, Wilkinson JL (1976). Congenitally corrected transposition of the great arteries morphologic study of 32 cases. Am J Cardiol.

[14] Kumar S, Phadke M, Kerkar P (2013). Supratricuspid obstructive membrane in congenitally corrected transposition of the great arteries. Ann Pediatr Cardiol.

[15] Henning RJ (2022). Tricuspid valve regurgitation current diagnosis and treatment. Am J Cardiovasc Dis.

